# Silencing of core transcription factors in human EC cells highlights the importance of autocrine FGF signaling for self-renewal

**DOI:** 10.1186/1471-213X-7-46

**Published:** 2007-05-16

**Authors:** Boris Greber, Hans Lehrach, James Adjaye

**Affiliations:** 1 Max Planck Institute for Molecular Genetics, Department of Vertebrate Genomics, 14195 Berlin, Germany

## Abstract

**Background:**

Despite their distinct origins, human embryonic stem (hES) and embryonic carcinoma (hEC) cells share a number of similarities such as surface antigen expression, growth characteristics, the ability to either self-renew or differentiate, and control of the undifferentiated state by the same core transcription factors. To obtain further insights into the regulation of self-renewal, we have silenced hES/hEC cell-specific genes in NCCIT hEC cells and analysed the downstream effects by means of microarrays.

**Results:**

RNAi-mediated silencing of OCT4 and SOX2 induced differentiation with mesodermal characteristics. Markers of trophoblast induction were only transiently up-regulated in the OCT4 knock-down. Independent knock-downs of NANOG produced a proliferation rather than a differentiation phenotype, which may be due to high NANOG expression levels in the cell line used. Published ChIP-chip data from hES cells were used to identify putative direct targets. RNAi-mediated differentiation was accompanied by direct down-regulation of known hES/hEC cell markers. This included all three core transcription factors in the case of the OCT4 and SOX2 knock-downs, confirming previous findings of reciprocal activation in ES cells. Furthermore, large numbers of histone genes as well as epigenetic regulators were differentially expressed, pointing at chromatin remodeling as an additional regulatory level in the differentiation process. Moreover, loss of self-renewal was accompanied by the down-regulation of genes involved in FGF signaling. FGF receptor inhibition for short and prolonged periods of time revealed that the ERK/MAPK cascade is activated by endogenously expressed fibroblast growth factors and that FGF signaling is cruicial for maintaining the undifferentiated state of hEC cells, like in hES cells.

**Conclusion:**

Control of self-renewal appears to be very similar in hEC and hES cells. This is supported by large numbers of common transcription factor targets and the requirement for autocrine FGF signaling.

## Background

Human embryonic stem cells (hESCs) are, like mouse ES cells, derived from the inner cell mass of blastocyst-stage embryos and capable of differentiating along the three germ layer lineages *in vivo *and *in vitro *[1,2]. Another defining property of ES cells is their ablity to self-renew under appropriate conditions, i.e. to give rise to equivalent daughter cells allowing indefinite propagation in culture [3]. The undifferentiated state is maintained by the action of transcription factors (TFs) some of which are ES cell-specific and common between human and mouse ES cells [4-7]. Boyer et al. [8] have identified binding sites of the core transcription factors OCT4, NANOG, and SOX2 within regulatory regions of most known genes by ChIP-chip analyses using human ES cells. This landmark study revealed that these three factors bind to large numbers of both transcribed and inactive genes many of which are co-occupied by at least two of these three factors. These included the *OCT4*, *NANOG*, and *SOX2 *genes themselves, thus suggesting auto and reciprocal regulation amongst themselves, which is supported by independent findings [9-11]. A number of target genes inactive in hES cells were developmental regulators, thus suggesting that the core transcription factors maintain the undifferentiated state by directly repressing these. Another level of transciptional repression has recently been revealed by genome-wide location analyses of Polycomb complexes which repress their target genes through epigenetic modification of chromatin structure [12,13]. ES cell chromatin is characterised by an overall decondensed structure, active histone marks and a large fraction of only loosely bound proteins, notably histones. Furthermore, chromatin remodelling factors may actively contribute to stem cell maintenance and early differentiation [[14], and reviewed in [15]].

Despite these similarities between human and mouse ES cells there are striking differences with regards to surface marker expression, signaling pathways promoting self-renewal [16-19], and overall growth properties. For instance, mouse ES cells are positive for the cell surface antigen SSEA1 but not for SSEA3 and 4. Conversely, hESCs are negative for SSEA1 but stain positive for SSEA3 and 4 [20]. In hES cells, activation of SMAD 2/3 by TGFβ/Activin/Nodal (TGFβ pathway) and suppression of BMP signaling is required for the maintenance of the undifferentiated state, in Serum Replacement-containing medium [21-24]. Moreover, FGF signaling appears to fulfill a master regulatory role in sustaining hESC self-renewal since these cells can be propagated under chemically defined conditions with bFGF supplementation only [25]. Furthermore, unlike mESCs, human ES cells display very low replating efficiencies with high rates of spontaneous differentiation when seeded as individual cells [26]. Thus, they need to be passaged as clumps of cells, which also enhances their karyotypic stability [27].

RNA interference [28] is a powerful tool to investigate self-renewal in ES cells [29]. Unfortunately, human ES cells are inefficiently transfected using conventional lipofection protocols. However, human embryonic carcinoma cells may present an alternative model system, as recently proposed [30]. These are the stem cells of teratocarcinomas, a subset of germ cell tumors. Despite their distinct origin, hEC cells share a number of characteristics with hES cells such as cellular morphology, surface antigen expression, differentiation capacity, and control of the undifferentiated state by OCT4 [31-33]. In addition, the overall gene expression profiles of hEC and hES cells appear to be very similar [[34,35], and Additional file 1]. Moreover, most hEC cell lines do not require special culture conditions such as feeder layer support or the addition of extrinsic factors. This, however, may be due to their aneuploid nature resulting in part from an adaptation to the culture environment. In fact, hES cells tend to acquire similar chromosomal abnormalities as hEC cells do [34,36,37]. Moreover, the need to maintain most hEC cell lines at high density [31] may be an indication for autocrine activation of signaling pathways sustaining self-renewal. However, this remains to be investigated.

For these reasons, hEC cells may be a suitable model for studying the regulation of self-renewal in pluripotent human cells. Results obtained from such an analysis may be extrapolated to hES cells or reveal specific differences between the two types of cells.

## Results and discussion

### Silencing of gene products enriched in hEC and hES cells

**Figure 1:**
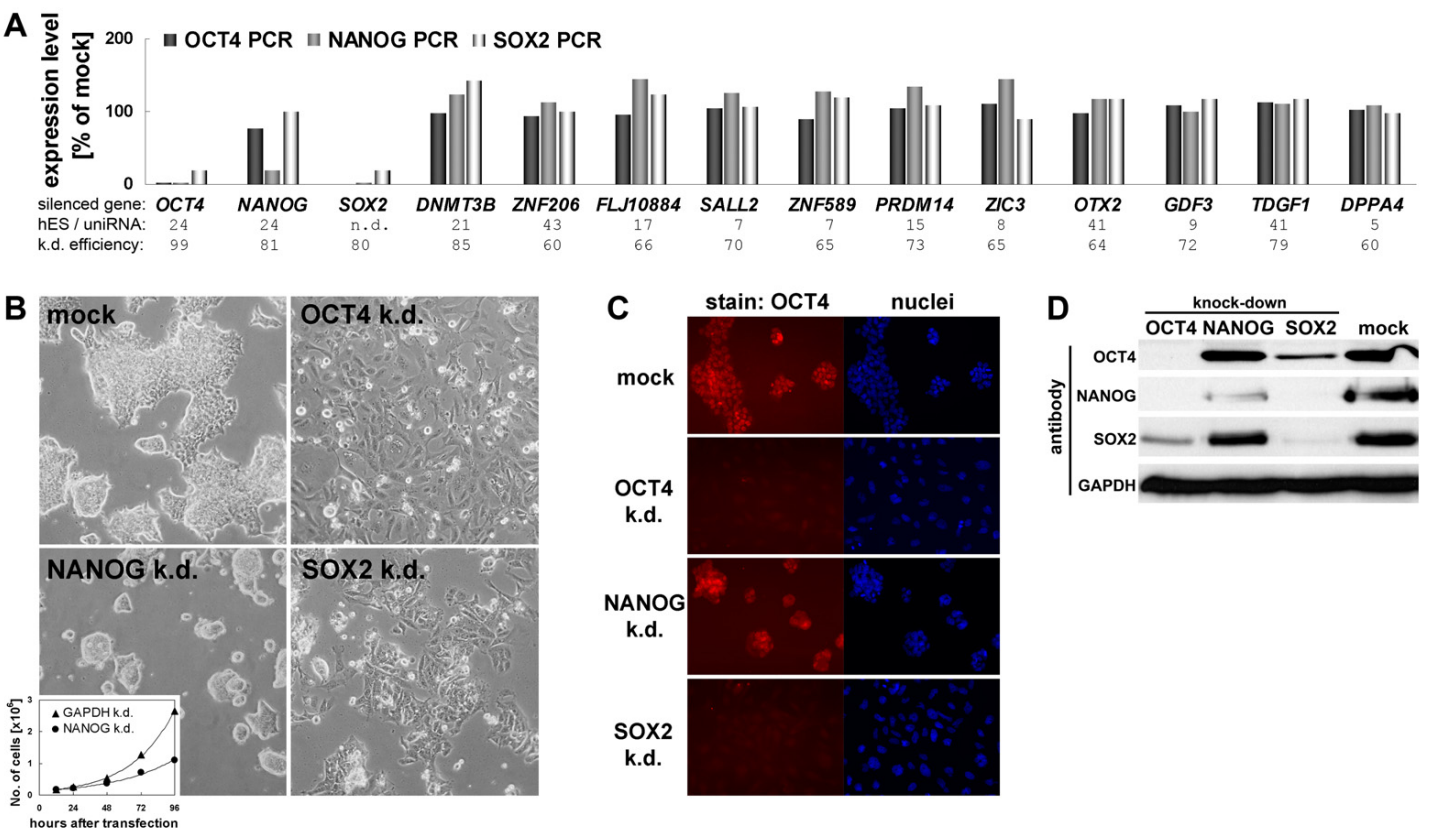
Silencing of OCT4, NANOG, and SOX2 in hEC cells. (A): Initial RNAi screen on hESC marker genes in hEC cells. esiRNA-treated samples were evaluated on the basis of cell morphology and changes in *OCT4*, *NANOG*, and *SOX2 *expression levels (by real-time PCR). Numbers at the bottom are array-based expression ratios of hESCs vs. universal reference RNA. The values for *GDF3 *and *OTX2 *are from an in-house platform (our unpublished data) and [34], respectively. Knock-down efficiencies were between 60 and > 90% throughout (not shown). (B): RNAi phenotypes in the OCT4, NANOG, and SOX2 knock-downs. Pictures were taken 2.5 days after esiRNA transfection. The morphology of unmanipulated or mock-treated cells was dependent on the seeding density. When plated at low density as required for esiRNA transfections the cells grew as 3D-shaped colonies rather than in monolayers. Bottom left: Growth curves of NANOG vs. GAPDH esiRNA-transfected cells. (C): Immunostaining of OCT4 protein in samples prepared as in (B). Note that the NANOG RNAi cells are OCT4 positive. (D): Western blot on day 3 RNAi and mock control samples using OCT4, NANOG, and SOX2 antibodies. GAPDH served as a loading control.

Genes controling the maintenance of the undifferentiated state in hES cells may be ES cell-specific and/or down-regulated upon differentiation. Based on these requirements we performed whole genome expression analysis of hES cells and compared these samples to universal reference RNA of pooled cell lines (Additional file 1). This resulted in the identification of > 1000 genes that are preferentially expressed in hESCs (ratio > 3). To further filter the dataset we used expression data of undifferentiated vs. differentiated hES cells and work published by others [4,34,38-43]. We then defined a set of genes supported by a maximum number of these datasets to be enriched in undifferentiated hESCs, regardless of their putative functions. A subset of these genes was confirmed to be also expressed in hEC cells and selected for RNAi-mediated knock-down. We monitored the effects of these perturbations by morphological criteria as well as measurements of *OCT4*, *NANOG*, and *SOX2 *expression levels (Fig. 1A). The majority of knock-downs did not reveal apparent morphological changes and displayed normal mRNA levels of the core transcription factors. This suggests that these genes do not have master regulatory roles in maintaining the undifferentiated state, while we cannot rule out insufficient silencing efficiencies as an alternative explanation for our observations in some cases (Fig. 1A). Silencing of OCT4 and SOX2 produced apparent morphological changes in that cells treated with OCT4 and SOX2 esiRNA [44] differentiated from 1–2 days post transfection.

The NANOG knock-down, somewhat surprisingly, yielded a diminished proliferation phenotype (Fig. 1B). In contrast, in human ES cells silencing of NANOG has been shown to result in the loss of pluripotency [45,46]. To confirm the NANOG RNAi phenotype in our hEC cells we employed a second esiRNA pool corresponding to a different region of the transcript. Again, we observed a significant reduction in cellular proliferation (not shown). Moreover, we did not observe this in any other knock-down performed, thus suggesting that the phenotype is specific. In contrast to the OCT4 and SOX2 RNAi samples, cells treated with NANOG esiRNA remained OCT4 positive, as monitored by immunocytochemistry (Fig. 1C). Western blot analysis showed that SOX2 protein levels also remained unaffected in the NANOG knock-down. In contrast, silencing of OCT4 also caused strong down-regulation of SOX2 and diminished NANOG to levels below those in the NANOG knock-down itself. Likewise, silencing of SOX2 caused substantial reduction of both OCT4 and NANOG protein (Fig. 1D). With the exception of the NANOG knock-down these data are in agreement with the model of auto and reciprocal regulation between the three transcription factors [8-11]. To investigate whether the failure of NANOG silencing in reducing OCT4 and SOX2 levels points at a bona fide biological difference between hEC and hES cells or to a specific characteristic of the hEC cell line used [47], we employed a different batch of NCCIT cells (ATCC CRL-2073). Again, we observed somewhat reduced proliferation but no differentiation following the silencing of NANOG, regardless if using a single esiRNA pool or a combination of esiRNAs 1 and 2 (Additional file 2A). In contrast, in lines 2102Ep and NTERA2 OCT4 and SOX2 levels were altered and *CDX2*, a marker of trophoblast induction [48], was up-regulated. However, while 2102Ep cells completely differentiated towards a TE-like phenotype with prominent nuclei, NTERA2 cells only showed partial differentiation and modest induction of later trophoblast markers *KRT7 *[18] and *HCG*. Hence, there appears to be heterogeneity between available hEC cell lines, whereas the NANOG RNAi phenotype in hES cells may probably be best mimicked with the 2102Ep line [30,45,46]. However, due to OCT4 and SOX2 levels being unchanged in the NANOG knock-down (Fig. 1D), we reasoned that the NCCIT line may be best suited to reveal NANOG-dependent genes on a global scale (see below).

As a possible explanation for the failure of NCCIT cells to differentiate following the silencing of NANOG, we considered that the NANOG protein level in the RNAi samples may still be sufficiently high to sustain self-renewal. This may seem likely given that chromosome 12 (where *NANOG *is located) is frequently amplified in hEC cells as compared to karyotypically normal hES cells [34] and because the NANOG knock-down was incomplete (Fig. 1D). Hence, NANOG protein levels in esiRNA-treated and mock-transfected NCCIT cells were compared to that in undifferentiated hES cells grown in a chemically defined medium [25]. Indeed, it appeared that NANOG is substantially more abundant in NCCIT cells as compared to hES cells and that the level after knock-down is still in the range of that in self-renewing hES cells (Additional file 3).

### Global expression analysis

**Figure 2:**
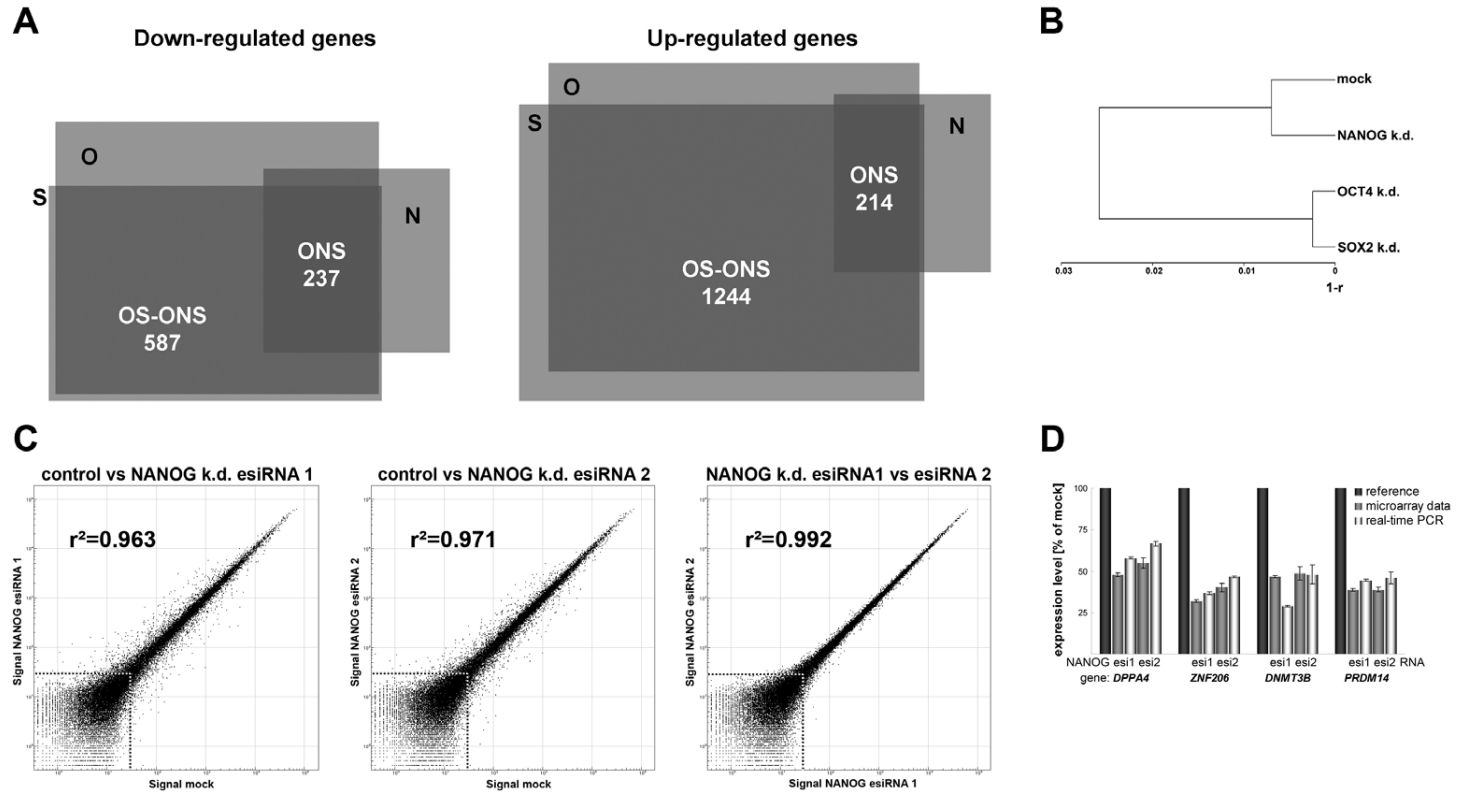
Expression profiling of OCT4, NANOG, and SOX2 RNAi samples. (A): Overlaps of differentially expressed genes. The areas of the squares are proportional to the numbers of genes which they represent [77]. O = OCT4 knock-down, N = NANOG k.d., S = SOX2 k.d. (B): Correlation-based dendogram of RNAi samples and control. Note the high similarity between the OCT4 and SOX2 knock-downs. r = linear correlation coefficient. (C): Scatter plots of independent NANOG knock-downs and mock controls. esiRNA 1 and 2 were derived from non-overlapping parts of the NANOG mRNA. The dashed line indicates the expression threshold based upon negative control beads. Only significantly expressed genes were considered in the correlation analysis. (D): Array and real-time PCR-based mRNA expression measurements of NANOG downstream targets using the two independent NANOG esiRNA pools. Note the high degree of concordance between the independent knock-downs and methods of mRNA quantification. Values are means of two biological replicates ± SE.

To reveal the overall downstream effects of the individual perturbations, we performed whole genome expression analysis on a quantitative microarray platform [49]. Using stringent filtering criteria revealed large numbers of differentially expressed genes which are presented as supplementary information (Additional file 4 A–F). The numbers, as expected given the vast change in cellular identity (Fig. 1B), were higher in the OCT4 and SOX2 knock-downs. There were substantial overlaps between the sets of up- and down-regulated genes, in particular between the OCT4 and SOX2 knock-downs (Fig. 2A). Consequenty, a comparison between the overall transcriptomes revealed that the OCT4 and SOX2 RNAi samples were highly similar to each other, while the NANOG knock-down was more closely related to the control cells (Fig. 2B). The non-overlapping parts regarding the OCT4 and SOX2 RNAi samples (light grey areas in Fig. 2A) arose largely due to the arbitrary thresholds set to identify differential gene expression. For instance, most genes seemingly down-regulated exclusively in the OCT4 knock-down were also down-regulated upon silencing of SOX2, albeit to a lesser extent (ratio > 0.67) or the changes were statistically less significant. Hence, the OCT4 and SOX2 RNAi samples were more or less equivalent and could therefore serve as specificity controls for one another. This was also confirmed by real-time PCR for a subset of the differentially expressed genes (see Additional file 5). To address the issue of off-target effects [50] in the NANOG knock-down we additionally analysed RNAi samples produced with an alternative esiRNA amplicon. This revealed a remarkable similarity between the independent RNAi samples in that essentially the same genes were identified as being differentially expressed (Fig. 2C). An explanation for the reduction of off-target effects using pooled siRNAs has been proposed [51]. In conclusion, the false positive rate of the data (Additional file 4) is low. To test whether the expression changes measured using the microarrays are quantitative, we additionally determined the expression ratios of several NANOG targets using real-time RT-PCR. This revealed a strong agreement between independent knock-downs using different esiRNAs and the two methods for transcript level quantification (Fig. 2D). This, together with data presented in Additional file 5, suggests that the microarray data are indeed quantitative.

### Expression of lineage-specific markers

The cells obtained after silencing of OCT4 and SOX2 had a flattened but overall undefined morphology accompanied by a reduction of cellular proliferation (Fig. 1B). To characterise the nature of the differentiated cells we investigated the expression of lineage-specific markers. Suppression of OCT4 activity in hES cells results in the up-regulation trophectodermal and primitive endoderm markers [32,42,52]. Similarly, several, but not all, hEC cell lines have been shown by PCR to up-regulate markers of the trophoblast lineage following OCT4 knock-down [32,33]. We did not detect trophoblast-specifying genes such as *CDX2 *in the OCT4 RNAi samples, by means of microarrays. To confirm this, we used real-time PCR as a more sensitive means of quantifying changes in gene expression, employing two different batches of NCCIT cells as well as other hEC cell lines. In 2102Ep and, somewhat surprisingly [32], NTERA2 cells, silencing of OCT4 lead to the up-regulation of all trophoblast differentiation markers tested (CDX2, KRT7 – data in [18], and HCG). However, the NCCIT cells only showed a transient induction of CDX2 and KRT7 at day 2 but no significant induction of the later marker HCG at day 4 (Additional file 2A). Hence, the NCCIT cells did not differentiate to trophoblast following OCT4 knock-down.

Instead, from a collection of marker gene expression data, it was evident that they have mesodermal characteristics. For instance, we observed strong induction of regulators such as *MSX1*, *HAND1*, and *RUNX1*, morphogens (*BMP4*), smooth muscle and cardiac actin, as well as mesoderm-specific collagens. Notably, most of these markers were not up-regulated in the NANOG knock-down, consistent with its undifferentiated phenotype. In contrast, virtually no endodermal and ectodermal marker genes were induced (Table 1). Since the original NCCIT cells had been shown to be capable of extraembryonic differentiation [47,53], the failure to continuously up-regulate trophectodermal markers may be due to a partial loss of developmental potential at some point in the past. Alternatively, it may be a cell type-specific effect. Interestingly, there are indications that trophoblast and mesodermal differentiation programmes may be somewhat related in that they involve at least some shared players. For instance, the transcription factor EOMES is expressed in both lineages and required for their specification [54]. Also, HAND1 has been implicated in trophoblast differentiation as well as in extraembryonic mesoderm and cardiac development [55,56]. Moreover, while bone morphogenic proteins are known inducers of mesodermal gene expression in different systems including hEC cells [31], BMP4 in particular has also been shown to promote trophoblast differentiation of hES cells [18]. We therefore speculate that the mesoderm-like differentiation in NCCIT cells may be a default fate aquired upon OCT4 ablation, possibly mediated by the induction of BMPs that could otherwise be interpreted as trophoblast-inducing signals (see below).

**Table 1:** Array-based expression ratios of lineage-specific markers in the OCT4, NANOG, and SOX2 knock-downs

**Lineage**	**Ratio**	**Ratio**	**Ratio**
*/GENE*	OCT4 kd	NANOG kd	SOX2 kd
**endoderm**			
*AFP*	n/a	n/a	n/a
*FGF8*	n/a	n/a	n/a
*GATA4*	n/a	n/a	n/a
*GCK*	n/a	n/a	n/a
*HNF4A*	n/a	n/a	n/a
*ONECUT1*	n/a	n/a	n/a
**ectoderm**			
*FGF5*	n/a	n/a	n/a
*HOXB1*	n/a	n/a	n/a
*LHX5*	2.9**	**1.0**	1.7*
*NEUROD1*	n/a	n/a	n/a
*NOG*	n/a	n/a	n/a
*OLIG2*	n/a	n/a	n/a
*OTX1*	n/a	n/a	n/a
*PAX6*	n/a	n/a	n/a
*SOX1*	n/a	n/a	n/a
**mesoderm**			
*ACTA2*	22.6**	n/a	22.6**
*ACTC*	9.2**	n/a	9.2**
*BMP4*	5.9**	**1.1**	5.3**
*CD34*	n/a	n/a	n/a
*COL1A1*	3.2**	n/a	4.4**
*COL2A1*	8.4**	n/a	8.0**
*HAND1*	9.1**	3.8**	8.1**
*MSX1*	4.9**	n/a	4.8**
*MYOD1*	n/a	n/a	n/a
*RUNX1*	5.3**	n/a	5.5**
*T*	n/a	n/a	n/a
**trophoblast**			
*CDX2*	n/a	n/a	n/a
*CSH1*	n/a	n/a	n/a
*HCG*	n/a	n/a	n/a
*KRT7*	n/a	n/a	n/a
**prim. endoderm**			
*GATA6*	**1.4**	n/a	**1.3**

With the exception of *LHX5*, *BMP4*, and *HAND1*, none of the genes was expressed at detectable levels in mock-treated hEC cell cultures. We did not investigate whether *T *was transiently up-regulated during cellular differentiation. For the calculation of expression ratios reference intensities were arbitrarily set to the level of threshold expression. n/a denotes insignificant expression also in the RNAi samples. * and ** indicate statistical significance of the expression changes (P < 0.05 and < 0.01, respectively).

### Downstream targets of OCT4, NANOG, and SOX2 in hECCs

Clearly, the large numbers of differentially expressed genes following OCT4, NANOG, and SOX2 RNAi (Fig. 2A, Additional file 4) are only in part directly regulated by these factors. To reveal which of the differentially expressed genes may be putative direct targets we made use of the ChIP-chip dataset by Boyer et al. [8] which is based on hES cells. Overall, there was a substantial overlap between the different datasets, which, however, was reduced when applying high-stringency filtering criteria for differential expression to our RNAi-chip data (Additional file 4). For instance, of the 414 genes down-regulated upon silencing of NANOG, 66 are direct targets in hES cells, based on the Boyer dataset. Significantly, most of these were also down-regulated in the OCT4 and SOX2 knock-downs, as expected, because in these samples NANOG was diminished, as well (Fig. 1A and 1D, left part of Fig. 2A – "ONS"). These genes are therefore likely to be NANOG targets directly activated both in undifferentiated hES and hEC cells. Examples include genes preferentially expressed in hES cells [4,34,38] such as *DPPA4*, *GDF3*, *HESX1*, *PRDM14*, *TERF1*, *ZIC2*, and *ZNF206 *(Table 2). Some of these as well as genes indirectly down-regulated in all three knock-downs (*DNMT3B*) were part of our initial screen (Fig. 1A). The fact that these genes were also down-regulated in the NANOG RNAi samples, which did not show any signs of differentiation, might explain why their silencing did not result in a loss of the undifferentiated state and, hence, they may not be absolutely required for self-renewal. Therefore, in the context of hES and hEC self-renewal, genes down-regulated in the OCT4 and SOX2 but not in the NANOG knock-downs are of utmost interest (left part of Fig. 2A – "OS-ONS"). This set comprises 145 genes directly regulated by either of the three TFs. Many of these as well as indirectly affected genes are also markers of undifferentiated ES cells. Examples include *AASS*, *DPPA3 *(*STELLA*), *FLJ10884*, *FOXO1A*, *LIN28*, *POU5F1 *(encoding OCT4) itself, *SOX8*, *SOX13*, *STAT3 *[57], *TDGF1*, *TRIM22*, *ZIC3*, *ZNF589*, and, interestingly, kruppel-like factor 4 (*KLF4*, Table 2). Klf4 has recently been implicated in reprogramming fibroblasts to an ES cell-like state, in conjuction with Oct4, Sox2, and c-Myc [58]. Hence, the orthologue *KLF4 *may play an important role in hEC and hES cells, as well. The mRNA level of *MYC*, though expressed in hEC and hES cells, did not change significantly in the OCT4 and SOX2 knock-downs (data not shown). Another gene, *Esrrb *(estrogen-related receptor beta), has recently been identified as a key regulator in mouse ES cells by two independent studies [29,59]. However, based on our array data, the expression of the orthologue *ESRRB *was below detectable levels both in hEC and hES cells.

**Table 2:** Examples of differentially expressed genes.

		**hEC cells**			**hES cells**			
		
**Symbol**	**Gene Name**	**Ratio RNAi/mock**			**ChIP-chip data [8]**			**Ratio**
**down-regulated genes**		**OCT4**	**NANOG**	**SOX2**	**OCT4**	**NANOG**	**SOX2**	**hES/uni**

*DPPA4*	developmental pluripotency associated 4	**0.1**	**0.4**	**0.1**	+	+	+	5
*ERBB2*	v-erb-b2 erythroblastic leukemia viral oncogene homolog 2	**0.6**	**2.1**	**0.4**	-	+	+	8
*FEZ1*	fasciculation and elongation protein zeta 1	**0.1**	**3.2**	**0.1**	+	+	+	6
*FLJ10884*	hypothetical protein FLJ10884	**0.3**	0.8	**0.3**	-	+	+	17
*FOXO1A*	forkhead box O1A	**0.5**	1.1	**0.5**	+	+	+	9
*GDF3*	growth differentiation factor 3	**0.0**	**0.4**	**0.0**	-	+	-	
*HESX1*	homeo box 1	**0.2**	**0.2**	**0.2**	+	+	+	5
*H2AFX*	H2A histone family, member X	**0.6**	1.0	**0.6**	-	+	+	
*HIST1H2BE*	histone 1, H2be	**0.6**	1.0	**0.6**	+	+	+	
*HIST1H2BG*	histone 1, H2bg	**0.6**	0.7	**0.5**	-	+	+	
*HIST1H2BH*	histone 1, H2bh	**0.5**	0.9	**0.5**	-	+	+	
*HIST1H2BJ*	histone 1, H2bj	**0.4**	0.9	**0.4**	-	-	+	
*HIST1H3D*	histone 1, H3d	**0.4**	0.8	**0.4**	+	+	+	
*HIST1H3F*	histone 1, H3f	**0.4**	0.7	**0.4**	-	+	+	
*HIST1H3J*	histone 1, H3j	**0.5**	**0.6**	**0.5**	+	+	+	
*HIST2H2AC*	histone 2, H2ac	**0.6**	1.0	**0.6**	+	-	+	
*HIST2H2BE*	histone 2, H2be	**0.5**	0.7	**0.5**	+	-	+	
*HIST2H3C*	histone 2, H3c	**0.2**	**0.6**	**0.2**	-	+	+	
*HIST2H4*	histone 2, H4	**0.3**	0.7	**0.3**	+	+	+	
*HIST3H2A*	histone 3, H2a	**0.3**	1.0	**0.3**	-	+	+	
*NANOG*	Nanog homeobox	**0.1**	**0.2**	**0.1**	+	+	+	24
*POU5F1*	POU domain, class 5, transcription factor 1	**0.0**	0.7	**0.0**	+	+	+	24
*PRDM14*	PR domain containing 14	**0.2**	**0.3**	**0.2**	+	+	+	15
*SMARCA3*	SWI/SNF related [...] regulator of chromatin [...]	**0.5**	**0.5**	**0.5**	-	+	+	
*STAT3*	signal transducer and activator of transcription 3	**0.6**	1.5	**0.6**	+	+	+	
*TDGF1*	teratocarcinoma-derived growth factor 1	**0.1**	0.7	**0.1**	+	+	+	41
*TERF1*	telomeric repeat binding factor 1	**0.2**	**0.3**	**0.2**	-	+	-	18
*TRIM22*	tripartite motif-containing 22	**0.3**	1.3	**0.3**	+	+	+	5
*ZIC2*	Zic family member 2	**0.4**	**0.5**	**0.4**	+	+	+	5
*ZIC3*	Zic family member 3 heterotaxy 1	**0.3**	1.0	**0.3**	+	+	+	8
*ZNF206*	zinc finger protein 206	**0.0**	**0.3**	**0.0**	-	+	-	43
*AASS*	aminoadipate-semialdehyde synthase	**0.6**	0.8	**0.5**				12
*DNMT3B*	DNA-methyltransferase 3 beta	**0.2**	**0.6**	**0.2**				21
*DPPA3*	developmental pluripotency associated 3 (STELLA)	**0.1**	0.7	**0.0**				
*KLF4*	Kruppel-like factor 4	**0.2**	0.8	**0.2**				
*LIN28*	lin-28 homolog	**0.6**	1.0	**0.7**				9
*MYST2*	MYST histone acetyltransferase 2	**0.3**	**0.6**	**0.3**				4
*SOX13*	SRY-box 13	**0.4**	1.1	**0.4**				4
*SOX8*	SRY-box 8	**0.2**	0.9	**0.2**				5
*THY1*	Thy-1 cell surface antigen	**0.2**	**0.3**	**0.1**				6
*UTF1*	undifferentiated embryonic cell transcription factor 1	**0.1**	**0.5**	**0.0**				26
*ZNF589*	zinc finger protein 589	**0.5**	1.0	**0.5**				7
**up-regulated genes**								

*BMP7*	bone morphogenetic protein 7	**2.7**	0.9	**3.0**	+	+	+	
*LEFTA*	left-right determination, factor A	**50.6**	**6.6**	**54.6**	+	+	+	4
*HAND1*	heart and neural crest derivatives expressed 1	**9.1**	**3.8**	**8.1**	+	+	+	
*HDAC9*	histone deacetylase 9, transcript variant 3	**2.3**	1.0	**2.1**	-	-	+	
*JUN*	v-jun sarcoma virus 17 oncogene homolog	**7.6**	**2.0**	**9.4**	-	+	-	
*KRT18*	keratin 18	**2.9**	**0.5**	**3.0**	-	+	-	
*MEIS2*	Meis1, myeloid ecotropic viral integration site 1 homolog 2	**2.5**	**2.1**	**2.8**	+	-	-	
*ATBF1*	AT-binding transcription factor 1	**2.2**	**2.3**	**2.3**	+	+	+	
*DLX1*	distal-less homeo box 1	**4.1**	1.0	**4.0**	+	+	+	
*DLX2*	distal-less homeo box 2	**9.3**	1.3	**9.1**	+	-	-	
*ISL1*	ISL1 transcription factor, LIM/homeodomain	**9.0**	0.7	**7.2**	+	+	+	
*LHX1*	LIM homeobox 1	**2.0**	1.0	**1.5**	+	+	-	
*LHX2*	LIM homeobox 2	**5.8**	0.9	**6.2**	+	+	+	
*LHX5*	LIM homeobox 5	**2.9**	1.0	**1.7**	+	+	+	
*PHTF2*	putative homeodomain transcription factor 2	**1.7**	1.3	**1.8**	-	+	-	
*ZFHX1B*	zinc finger homeobox 1b	**1.8**	**1.8**	**2.2**	+	+	+	
*DLX3*	distal-less homeo box 3	**3.1**	1.3	**2.9**				
*IRX3*	iroquois homeobox protein 3	**4.0**	1.0	**3.7**				
*IRX4*	iroquois homeobox protein 4	**2.5**	1.0	**2.6**				4
*MSX1*	msh homeo box homolog 1	**4.9**	1.0	**4.8**				
*MSX2*	msh homeo box homolog 2	**1.6**	**0.6**	**1.6**				
*PKNOX2*	PBX/knotted 1 homeobox 2	**4.9**	**2.5**	**5.9**				
*PRRX2*	paired related homeobox 2	**1.9**	**1.8**	**1.8**				
*ZHX1*	zinc fingers and homeoboxes 1	**3.1**	1.0	**3.3**				

Ratios > 1.5 or < 0.67 are in bold. In essentially all cases, these changes were also statistically significant. hESC ChIP-chip data were taken from [8]. The last column (expression ratio > 3 of H9 hES cells vs. universal reference RNA) indicates enriched gene expression in hES cells. SOX2 was below detection on the arrays but monitored by real-time PCR (Fig. 1A). See Additional file 5 for additional confirmations.

Furthermore, the genes down-regulated in the OCT4 and SOX2 but not in the NANOG RNAi samples comprised between ca. 20 to 30 histones. Depending on the thresholds for expression ratio and statistical significance, many of these are direct (SOX2) targets in hES cells, according to Boyer et al. [8] (Tables 2 and S1). In addition, we noticed that histone acetylases (*MYST2*, *MYST4*) as well as the SWI/SNF family member *SMARCA3 *were down-regulated and histone deacetylases (*HDAC5*, *HDAC9 *– direct SOX2 target in hES cells, Table 2) were induced. Besides the transcriptional control by TFs, these findings strongly suggest modification of chromatin structure as an additional mechanism to drive/accompany the transition from undifferentiated towards differentiated cell identity [42]. This is in agreement with the current model of chromatin states in ES vs. differentiated cells [15] and, maybe, with the discovery of hyperdynamic structural chromatin protein in undifferentiated ES and EC cells [14]. We also found the Polycomb group genes *PHC1 *(4-fold down) and *PHC2 *(4-fold up) to be differentially expressed in the OCT4 and SOX2 knock-downs. However, a comparison of our RNAi-chip data to PRC1 downstream targets in mouse ES cells [12] would go beyond the scope of this report and be too speculative at this point.

To further categorise the differentially expressed genes, with an emphasis on the OCT4 and SOX2 RNAi datasets, we mapped these to Gene Ontology terms [60]. As can be inferred from Table 3, the genes up-regulated after ablation of the core TFs were preferentially involved in intracellular signaling, differentiation, cytoskeletal organisation, apoptosis, Wnt pathway activation, cell cycle arrest, and cell adhesion/generation of extracellular matrix. These are all processes that one may predict to result from the extinction of self-renewal activity. Moreover, many induced genes are known to be involved in the generation of mesodermal cell types, confirming the analysis of marker gene expression (see above). Finally, transcription factors were enriched in the up-regulated gene sets indicating that these are repressed in the undifferentiated state and hence, may be involved in triggering the differentiation process or in maintaining the differentiated cell state. Some of them are known to be involved in lineage specification (Table 1). A large fraction was presented by homeobox-containing TFs many of which appear to be directly repressed by OCT4, NANOG, and/or SOX2 in hES cells [8] (Table 2).

**Table 3:** Selected Gene Ontology terms associated with genes differentially expressed in both the OCT4 and SOX2 knock-downs.

**Over-represented GO terms associated only with down-regulated gene sets**		
**Biological Process**	**No. of genes**	**P value**
primary metabolism	318	0.000
establishment and/or maintenance of chromatin architecture	23	0.000
mitotic cell cycle	14	0.024
steroid biosynthesis	9	0.004
activation of MAPK activity	5	0.026
**Molecular Function**	**No. of genes**	**P value**

catalytic activity	236	0.000
ATP binding	59	0.020
aminoacyl-tRNA ligase activity	9	0.000
GABA receptor activity	3	0.200
		
**Over-represented GO terms associated only with up-regulated gene sets**		
**Biological Process**	**No. of genes**	**P value**

intracellular signaling cascade	113	0.000
cell adhesion	92	0.000
apoptosis	68	0.000
cell differentiation	61	0.000
cytoskeleton organization and biogenesis	46	0.000
skeletal development	23	0.000
muscle development	20	0.000
Wnt receptor signaling pathway	16	0.002
cell cycle arrest	13	0.001
blood vessel development	11	0.013
**Molecular Function**	**No. of genes**	**P value**

calcium ion binding	94	0.000
transcription factor activity	85	0.000
cytoskeletal protein binding	63	0.000
protein kinase activity	58	0.002
extracellular matrix structural constituent	28	0.000

P values from the DAVID [76] output are a measure for the enrichment of the individual GO terms in the lists of up (1458) and down-regulated (824) genes.

In contrast, GO terms associated with the loss of self-renewal reflected typical ES/EC cell properties, namely high rates of primary metabolism (> 300 genes), cell cycle, ATP consumption, and protein biosynthesis (Table 3). The differential expression of genes involved in chromatin remodeling has been discussed above. However, the down-regulation of large numbers of histone genes might additionally be interpreted as simply reflecting the overall reduction of cell division. On the other hand, we did not see this tendency in the NANOG RNAi samples which also displayed reduced proliferation rates. The NANOG phenotype (Fig. 1B) is likely a result of a reduction in metabolic and cell cycle activity rather than from the induction of apoptosis, as suggested by GO and KEGG pathway analyses (results not shown). Of interest in this context may also be the significant down-regulation of *UTF1 *in the NANOG knock-down (Table 2) since the mouse orthologue has been implicated in ES cell proliferation [61] and revealed to be a direct Nanog target [59].

### Putative receptor-mediated signaling in hEC cells

To investigate whether there may be signaling pathways operative in maintaining the undifferentiated state of hEC cells, we mapped the sets of genes differentially expressed in the OCT4 and SOX2 knock-downs to known pathways [60]. We noticed that components of the TGFβ pathway were preferentially up-regulated upon differentiation (P < 0.01). These included *BMP1*, *4*, *6*, and *7*, *GREM1*, *TGFB2 *and *3*, *SMAD2 *and *3*, and – strikingly – *LEFTA *(> 50-fold up). *NODAL *and the antagonists *LEFTA *and *B *are expressed in hES cells and rapidly down-regulated upon differentiation [22,42]. In addition to Nodal [62], TGFβ1 and Activin A have been shown to support self-renewal of hES cells, by activating SMAD 2/3 [21,63-66]. However, the corresponding genes are not or only weakly expressed in hEC cells (Additional file 6) and, moreover, we failed to amplify the SMAD binding partner FOXH1 from hEC cDNA (not shown). These findings argue against a contribution of TGFβ signaling to self-renewal of NCCIT hEC cells, in contrast to the situation in hES cells when grown in Serum Replacement-based medium. Further investigation is required to test this hypothesis.

Nevertheless, there may also be similarities between hES and hEC cells with regards to (autocrine) receptor-mediated signaling. For instance, we noticed that 3 genes encoding GABA receptor components were down-regulated upon differentiation (Table 3). This is noteworthy because for example, *GABRB3 *(4-fold down in our OCT4 and SOX2 RNAi samples) is also enriched hESCs [34,35] and GABA as well as pipecolic acid have recently been found to support the undifferentiated growth of these cells [63].

**Figure 3:**
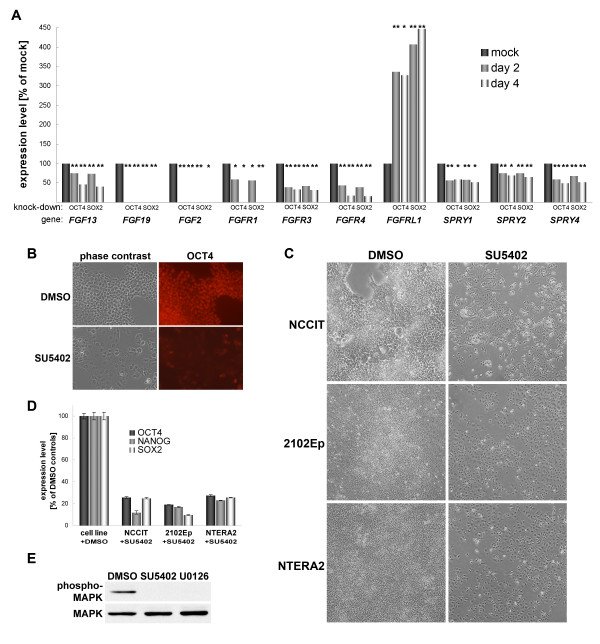
Autocrine FGF signaling is cruicial for hEC cell self-renewal. (A): Expression changes of genes involved in FGF signaling. Samples were assayed 2 and 4 days after transfection. Transcripts of *FGF19*, *FGF2*, and *FGFR1 *(at 96 h) were undetectable in the OCT4 and SOX2 RNAi samples. (B): Immunostain of OCT4 protein in NCCIT hEC cells 5 days after SU5402 treatment indicating loss of self-renewal. (C): Cellular morphology after 5 days of SU5402 treatment vs. DMSO controls using three different hEC cell lines. (D): Monitoring of *OCT4*, *NANOG*, and *SOX2 *expression levels by real-time PCR in samples from (C). Error bars indicate technical variation (DMSO control: means between cell lines). (E): Short-term effect of SU5402 treatment on MAPK phosphorylation in NCCIT hEC cells. Total MAPK protein (bottom panel) served as loading control. U0126 is a specific inhibitor of MEK (MAPKK) which directly phosphorylates ERK (MAPK). This sample served as positive control for the assay.

Moreover, we noticed that genes encoding MAPK-activating proteins were significantly down-regulated (Table 3, P < 0.05). Interestingly, these included fibroblast growth factors and FGF receptors. In addition, Sprouty genes which encode modulators of FGF/MAPK signaling induced by FGFs themselves [67,68] were repressed as well. In contrast, *FGFRL1*, encoding an FGF receptor devoid of an intracellular tyrosine kinase domain and therefore a putative inhibitor of FGF signaling, was strongly induced (Fig. 3A). To test whether FGF signaling is required for self-renewal of NCCIT cells, we cultured these in the presence and absence of a potent inhibitor of FGF receptors [69]. After one day of SU5402 treatment, the cells showed a reduction in growth rates and began to differentiate. After 5 days, the cells had essentially ceased to proliferate and were largely differentiated as monitored by OCT4 immunostaining (Fig. 3B). To rule out that these observations were specific to the NCCIT cells we employed two other hEC lines and obtained essentially the same results (Fig. 3C). OCT4, NANOG, and SOX2 levels were monitored by real-time PCR, which confirmed the loss of the undifferentiated state in all three cell lines. These data strongly supports the notion that FGF signaling is a general requirement for self-renewal of hEC cells. This is likely to happen in an autocrine manner since fetal calf serum contained in the culture medium does not contain FGFs or, at least, FGF2 [70]. A speculative hypothesis is that autocrine signaling provides an explanation for the fact that several hEC lines need to be cultured at high densities to prevent their spontaneous differentiation [31].

Interestingly, autocrine FGF signaling has also been shown to be crucial in human ES cells [71] and these also express FGF2, 13, and 19 which are down-regulated upon induction of differentiation [4,42]. While other pathways may as well be FGF-dependent in hES cells, FGF2 has been shown to activate the ERK/MAPK signaling cascade [72]. To test whether this is also the case in hEC cells, we monitored MAPK phosphorylation 30 minutes after SU5402 treatment. Indeed, MAPK was active in the control cells and its phosphorylation was abolished upon FGF receptor inhibition, as predicted (Fig. 3E).

## Conclusion

Overall, our data highlights the similarities between human EC and ES cells and further supports the idea that pluripotent human stem cells share a number of characteristics in the context of maintaining their cellular identity. This applies for the overall downstream targets of core transcription factors as well as for the requirement of autocrine FGF signaling. However, differences appear to exist with regards to the expression of TGFβ pathway genes.

## Methods

### esiRNA production

esiRNA was essentially produced as described elsewhere [73]. Briefly, T7-linked products from RT-PCRs on hEC cell cDNA were used as input for *in vitro *transcription reactions (Ambion). After annealing of the two RNA strands, long dsRNA was digested with in-house generated RNase III enzyme and purified to yield pools of siRNAs with an average size of 20–30 bp. 500–650 bp amplicons for the initial RT-PCRs were selected from repeat-masked cDNA regions following published guidelines to avoid theoretical cross-silencing [73]. Primer sequences are given in Additional file 7.

### Cell culture

hEC cells were grown in high-glucose DMEM supplemented with 10% FCS (Biochrom, Berlin/Germany), 2 mM glutamine, and penicillin/streptomycin on conventional tissue culture plastic surfaces. Different lines were compared with regards to their growth properties. We ultimately chose medium-passage NCCIT cells (a kind gift by Dr Leendert Looijenga) for our studies because this line showed the lowest rates of spontaneous differentiation when plated at low density (see Additional file 2C), as it is required for high-efficiency siRNA transfections. Knock-down efficiencies were optimised using esiRNA against GAPDH and lipofection reagents from different manufacturers. We then routinely transfected hECCs by seeding them at 20,000 cells/cm^2 ^along with esiRNA/Hyperfect (Qiagen) complexes (100 ng esiRNA/2 μl Hyperfect per cm^2^) in multiwell plates, following the "fast-forward" protocol by Qiagen. Treatment with either transfection reagent alone or Hyperfect plus irrelevant esiRNA did not significantly alter cell survival and growth rates. Silencing efficiencies with the GAPDH model esiRNA were ca. 90% under the optimised conditions. In experiments presented as supplementary data (Additional file 2), low-passage NCCIT cells directly obtained from ATCC (CRL-2073) were used in addition to the medium-passage NCCIT cells employed throughout this study. Proliferation curves were recorded by counting numbers of cells in GAPDH and NANOG esiRNA-transfected cultures by means of a hematocytometer. Cell numbers were normalised at the 12 h timepoint to account for slight variations in plating efficiencies. SU5402 (Calbiochem, 30 μM) was applied continuously to lines NCCIT, 2102Ep, and NTERA2 which were seeded at titres high enough to otherwise prevent significant spontaneous differentiation. DMSO-treated cultures served as controls. For short-term effects of SU5402 (30 μM) and U0126 (Promega, 20 μM) on MAPK phosphorylation semiconfluent NCCIT cultures were treated with these inhibitors and DMSO (as control) for 30 minutes. Representative morphology was recorded at 50-fold total magnification using an Olympus CK2 phase contrast microscope. RNA was isolated using Qiagen RNeasy kits with on-column DNase I digestion. Protein was extracted with an NP-40 and Benzonase-containing buffer.

H1 and H9 hES cells [1] were purchased from the WiCell Research Institute (Madison, Wisconsin), expanded on feeders, and adapted to feeder-free culture in MEF-conditioned medium for several passages before RNA isolation. hESCs were maintained undifferentiated in 6-wells pre-coated with Matrigel (BD Biosciences), using a combination of mechanical and enzymatic passaging. Composition of hES medium was as described elsewhere [74]. For experiments presented as supplementary data (Additional file 3), H9 hES cells were grown in a chemically defined medium [25] to minimise potential medium-dependent effects on NANOG protein levels.

### Real-time PCR, Western blotting, immunocytochemistry

RNA was reversely transcribed using MMLV (USB) and oligo-dT priming. Real-time RT-PCR was carried out on Applied Biosystems 7900 instrumentation in 20 μl reactions containing 10 μl of SYBR Green PCR mix (ABI), 0.375 μM of each primer, and diluted cDNA. All primer pairs used were confirmed to approximately double the amount of product within one cycle and to yield a single product of the predicted size. Primer sequences are provided in Additional file 7. Relative mRNA levels were calculated using the comparative Ct method (ABI instruction manual) and presented as % of biological controls. ACTB transcript levels were confirmed to correlate well with total RNA amounts and therefore used for normalisation throughout.

Western blotting was performed according to standard procedures and using chemiluminescence detection (ECL – Amersham). Antibodies used were Santa Cruz sc-8629 (OCT4), R&D AF1997 (NANOG), Santa Cruz sc-17320 X (SOX2), Ambion #4300 (GAPDH), Cell Signaling Technology #9102 and #9106 (p44/42 MAPK and phospho-MAPK, respectively), Calbiochem #401504 (HRP-linked), as well as Amersham NA9340 and NA9310 (HRP-linked).

Following paraformaldehyde fixation and permeabilisation with Triton X-100, OCT4 immunostaining was carried out employing antibodies sc-8629 (Santa Cruz) and A21468 (Amersham). Fluorescence was visualised using a Zeiss AxioImager Z1 microscope.

### Chip hybridisations and analysis of whole-genome expression data

Biotin-labelled cRNA was generated using a linear amplification kit (Ambion #IL1791) using 300 ng of DNA-free, quality-checked total RNA as input. Chip hybridisations, washing, Cy3-streptavidin (Amersham Biosciences) staining, and scanning was performed on an Illumina BeadStation 500 platform employing reagents and following protocols supplied by the manufacturer. cRNA samples were hybridised as biological triplicates on Illumina human-6 or human-8 BeadChips. Due to a 30-fold feature redundancy quantitative expression data can be obtained [49]. Samples to be hybridised were harvested 2 and 4 days after transfection. The OCT4 and SOX2 RNAi samples were morphologically indistinguishable in these experiments. Analysis of the data was focussed on the day 2 samples because the data of the two timepoints were found to be largely redundant (Fig. 3A). In addition to information provided in Additional file 4, day 2 processed and raw RNAi as well as hES cell expression data (vs. universal reference RNA – Stratagene #740000) have been made accessible through GEO [75] in a MIAME-compliant format (accession numbers GSE5422 and GSE5423).

All basic expression data analyses were carried out using the manufacturer's software BeadStudio 1.0. Raw data were background-subtracted and normalised using the "rank invariant" algorithm, by which negative intensity values may arise. These and values below the detection limit were arbitrarily set to the level of threshold detection (S = 20) in order to avoid nonsense values for expression ratios. Differentially expressed genes were required to change by at least 50% at P < 0.01 according to both the t-test (assuming equal variance) and an Illumina custom model [49]. Pathway and Gene Ontology analyses were carried out using DAVID 2006 [76]. In both cases, we used GenBank accession numbers represented by the corresponding chip oligonucleotides as input.

## Authors' contributions

BG conceived and carried out experiments, analysed data, and drafted the manuscript. HL provided materials the experimental platform essential to this work. JA conceived experiments, analysed data, and revised the manuscript. All authors read and approved the final manuscript.

## Supplementary Material

Additional file 1 Microarray-based transciptome comparison between hES cells and NCCIT hEC cells. Linear correlation coefficients (r) were 0.947 (NCCIT vs. H1), 0.934 (NCCIT vs. H9), and 0.984 (H1 vs. H9). Universal reference RNA (Stratagene) served as a baseline to identify genes preferentially expressed in hES and hEC cells. Click here for file

Additional file 2 Silencing of OCT4 and NANOG in 2102Ep, NTERA2, and two batches of NCCIT cells. (A) Real-time PCRs at two timepoints after esiRNA transfection using OCT4, NANOG, and SOX2 primers as well as markers of trophoblast differentiation. Bars indicate standard errors between two independent experiments (NCCIT) or between values based on distinct housekeeping controls (2102Ep and NTERA2). (B) Representative cellular morphology at day 4. See Fig. 3C for undifferentiated samples. (C) Real-time PCR-based comparison between cells kept undifferentiated by high density passaging vs. cells plated at low density as required for efficient gene silencing, using CDX2 as an early differentiation marker. Click here for file

Additional file 3 NANOG protein levels in NCCIT (with and without transfection of NANOG esiRNA) and H9 hES cells. Left: Western blot probed with NANOG and GAPDH antibodies. Right: Undifferentiated morphology of hES cells prior to harvesting. Click here for file

Additional file 4 Differentially expressed genes in the OCT4, NANOG, and SOX2 knock-downs. (A) up and (B) down in the OCT4 RNAi samples, (C) up and (D) down in the NANOG knock-down, (E) up and (F) down with silencing of SOX2. The expression of genes was required to change at least 1.5-fold at P < 0.01, according to two statistical tests. Specific gene expression in hES cells (ratio > 3 vs. universal RNA) and hESC ChIP-chip data from [8] are indicated in the last columns. Click here for file

Additional file 5 Real-time PCR confirmations of differential gene expression in the OCT4, NANOG, and SOX2 RNAi samples. (A) Relative mRNA levels as measured with microarrays (light columns) and real-time PCR (dark columns). Real-time PCR measurements are based on two housekeeping control genes (GAPDH and ACTB). (B) Correlation between array and real-time PCR data of part A. The slope and correlation coefficient given refer to those transcripts that could reliably be detected by the arrays in both conditions (slope with all data points: 1.2; R^2 ^= 0.88). Click here for file

Additional file 6 TGFβ pathway genes differentially expressed in the OCT4 and SOX2 knock-downs. *INHBA*, and *NODAL *were included because of their roles in sustaining the undifferenitated growth of hES cells, although their expression did not change significantly (n.s.) or was below detectable levels (n.expr.). The probe for *TGFB1 *was found to be falsely designed (n/a). Click here for file

Additional file 7 Primers for esiRNA production and real-time RT-PCR. Click here for file

## References

[B1] Thomson JA, Itskovitz-Eldor J, Shapiro SS, Waknitz MA, Swiergiel JJ, Marshall VS, Jones JM (1998). Embryonic stem cell lines derived from human blastocysts. Science.

[B2] Reubinoff BE, Pera MF, Fong CY, Trounson A, Bongso A (2000). Embryonic stem cell lines from human blastocysts: somatic differentiation in vitro. Nat Biotechnol.

[B3] Smith AG (2001). Embryo-derived stem cells: of mice and men. Annu Rev Cell Dev Biol.

[B4] Sato N, Sanjuan IM, Heke M, Uchida M, Naef F, Brivanlou AH (2003). Molecular signature of human embryonic stem cells and its comparison with the mouse. Dev Biol.

[B5] Mitsui K, Tokuzawa Y, Itoh H, Segawa K, Murakami M, Takahashi K, Maruyama M, Maeda M, Yamanaka S (2003). The homeoprotein Nanog is required for maintenance of pluripotency in mouse epiblast and ES cells. Cell.

[B6] Chambers I, Colby D, Robertson M, Nichols J, Lee S, Tweedie S, Smith A (2003). Functional expression cloning of Nanog, a pluripotency sustaining factor in embryonic stem cells. Cell.

[B7] Nichols J, Zevnik B, Anastassiadis K, Niwa H, Klewe-Nebenius D, Chambers I, Scholer H, Smith A (1998). Formation of pluripotent stem cells in the mammalian embryo depends on the POU transcription factor Oct4. Cell.

[B8] Boyer LA, Lee TI, Cole MF, Johnstone SE, Levine SS, Zucker JP, Guenther MG, Kumar RM, Murray HL, Jenner RG, Gifford DK, Melton DA, Jaenisch R, Young RA (2005). Core transcriptional regulatory circuitry in human embryonic stem cells. Cell.

[B9] Okumura-Nakanishi S, Saito M, Niwa H, Ishikawa F (2005). Oct-3/4 and Sox2 regulate Oct-3/4 gene in embryonic stem cells. J Biol Chem.

[B10] Chew JL, Loh YH, Zhang W, Chen X, Tam WL, Yeap LS, Li P, Ang YS, Lim B, Robson P, Ng HH (2005). Reciprocal transcriptional regulation of Pou5f1 and Sox2 via the Oct4/Sox2 complex in embryonic stem cells. Mol Cell Biol.

[B11] Rodda DJ, Chew JL, Lim LH, Loh YH, Wang B, Ng HH, Robson P (2005). Transcriptional regulation of nanog by OCT4 and SOX2. J Biol Chem.

[B12] Boyer LA, Plath K, Zeitlinger J, Brambrink T, Medeiros LA, Lee TI, Levine SS, Wernig M, Tajonar A, Ray MK, Bell GW, Otte AP, Vidal M, Gifford DK, Young RA, Jaenisch R (2006). Polycomb complexes repress developmental regulators in murine embryonic stem cells. Nature.

[B13] Lee TI, Jenner RG, Boyer LA, Guenther MG, Levine SS, Kumar RM, Chevalier B, Johnstone SE, Cole MF, Isono K, Koseki H, Fuchikami T, Abe K, Murray HL, Zucker JP, Yuan B, Bell GW, Herbolsheimer E, Hannett NM, Sun K, Odom DT, Otte AP, Volkert TL, Bartel DP, Melton DA, Gifford DK, Jaenisch R, Young RA (2006). Control of developmental regulators by Polycomb in human embryonic stem cells. Cell.

[B14] Meshorer E, Yellajoshula D, George E, Scambler PJ, Brown DT, Misteli T (2006). Hyperdynamic plasticity of chromatin proteins in pluripotent embryonic stem cells. Dev Cell.

[B15] Meshorer E, Misteli T (2006). Chromatin in pluripotent embryonic stem cells and differentiation. Nat Rev Mol Cell Biol.

[B16] Ying QL, Nichols J, Chambers I, Smith A (2003). BMP induction of Id proteins suppresses differentiation and sustains embryonic stem cell self-renewal in collaboration with STAT3. Cell.

[B17] Daheron L, Opitz SL, Zaehres H, Lensch WM, Andrews PW, Itskovitz-Eldor J, Daley GQ (2004). LIF/STAT3 signaling fails to maintain self-renewal of human embryonic stem cells. Stem Cells.

[B18] Xu RH, Chen X, Li DS, Li R, Addicks GC, Glennon C, Zwaka TP, Thomson JA (2002). BMP4 initiates human embryonic stem cell differentiation to trophoblast. Nat Biotechnol.

[B19] Pera MF, Andrade J, Houssami S, Reubinoff B, Trounson A, Stanley EG, Ward-van Oostwaard D, Mummery C (2004). Regulation of human embryonic stem cell differentiation by BMP-2 and its antagonist noggin. J Cell Sci.

[B20] Pera MF, Reubinoff B, Trounson A (2000). Human embryonic stem cells. J Cell Sci.

[B21] James D, Levine AJ, Besser D, Hemmati-Brivanlou A (2005). TGFbeta/activin/nodal signaling is necessary for the maintenance of pluripotency in human embryonic stem cells. Development.

[B22] Besser D (2004). Expression of nodal, lefty-a, and lefty-B in undifferentiated human embryonic stem cells requires activation of Smad2/3. J Biol Chem.

[B23] Xu RH, Peck RM, Li DS, Feng X, Ludwig T, Thomson JA (2005). Basic FGF and suppression of BMP signaling sustain undifferentiated proliferation of human ES cells. Nat Methods.

[B24] Wang G, Zhang H, Zhao Y, Li J, Cai J, Wang P, Meng S, Feng J, Miao C, Ding M, Li D, Deng H (2005). Noggin and bFGF cooperate to maintain the pluripotency of human embryonic stem cells in the absence of feeder layers. Biochem Biophys Res Commun.

[B25] Yao S, Chen S, Clark J, Hao E, Beattie GM, Hayek A, Ding S (2006). Long-term self-renewal and directed differentiation of human embryonic stem cells in chemically defined conditions. Proc Natl Acad Sci U S A.

[B26] Amit M, Carpenter MK, Inokuma MS, Chiu CP, Harris CP, Waknitz MA, Itskovitz-Eldor J, Thomson JA (2000). Clonally derived human embryonic stem cell lines maintain pluripotency and proliferative potential for prolonged periods of culture. Dev Biol.

[B27] Mitalipova MM, Rao RR, Hoyer DM, Johnson JA, Meisner LF, Jones KL, Dalton S, Stice SL (2005). Preserving the genetic integrity of human embryonic stem cells. Nat Biotechnol.

[B28] Elbashir SM, Harborth J, Lendeckel W, Yalcin A, Weber K, Tuschl T (2001). Duplexes of 21-nucleotide RNAs mediate RNA interference in cultured mammalian cells. Nature.

[B29] Ivanova N, Dobrin R, Lu R, Kotenko I, Levorse J, DeCoste C, Schafer X, Lun Y, Lemischka IR (2006). Dissecting self-renewal in stem cells with RNA interference. Nature.

[B30] Josephson R, Ording CJ, Liu Y, Shin S, Lakshmipathy U, Toumadje A, Love B, Chesnut JD, Andrews PW, Rao MS, Auerbach JM (2007). Qualification of embryonal carcinoma 2102Ep as a reference for human embryonic stem cell research. Stem Cells.

[B31] Andrews PW (2002). From teratocarcinomas to embryonic stem cells. Philos Trans R Soc Lond B Biol Sci.

[B32] Matin MM, Walsh JR, Gokhale PJ, Draper JS, Bahrami AR, Morton I, Moore HD, Andrews PW (2004). Specific knockdown of Oct4 and beta2-microglobulin expression by RNA interference in human embryonic stem cells and embryonic carcinoma cells. Stem Cells.

[B33] Andrews PW, Matin MM, Bahrami AR, Damjanov I, Gokhale P, Draper JS (2005). Embryonic stem (ES) cells and embryonal carcinoma (EC) cells: opposite sides of the same coin. Biochem Soc Trans.

[B34] Sperger JM, Chen X, Draper JS, Antosiewicz JE, Chon CH, Jones SB, Brooks JD, Andrews PW, Brown PO, Thomson JA (2003). Gene expression patterns in human embryonic stem cells and human pluripotent germ cell tumors. Proc Natl Acad Sci U S A.

[B35] Liu Y, Shin S, Zeng X, Zhan M, Gonzalez R, Mueller FJ, Schwartz CM, Xue H, Li H, Baker SC, Chudin E, Barker DL, McDaniel TK, Oeser S, Loring JF, Mattson MP, Rao MS (2006). Genome wide profiling of human embryonic stem cells (hESCs), their derivatives and embryonal carcinoma cells to develop base profiles of U.S. Federal government approved hESC lines. BMC Dev Biol.

[B36] Draper JS, Smith K, Gokhale P, Moore HD, Maltby E, Johnson J, Meisner L, Zwaka TP, Thomson JA, Andrews PW (2004). Recurrent gain of chromosomes 17q and 12 in cultured human embryonic stem cells. Nat Biotechnol.

[B37] Andrews PW (2006). The selfish stem cell. Nat Biotechnol.

[B38] Brandenberger R, Wei H, Zhang S, Lei S, Murage J, Fisk GJ, Li Y, Xu C, Fang R, Guegler K, Rao MS, Mandalam R, Lebkowski J, Stanton LW (2004). Transcriptome characterization elucidates signaling networks that control human ES cell growth and differentiation. Nat Biotechnol.

[B39] Bhattacharya B, Miura T, Brandenberger R, Mejido J, Luo Y, Yang AX, Joshi BH, Ginis I, Thies RS, Amit M, Lyons I, Condie BG, Itskovitz-Eldor J, Rao MS, Puri RK (2004). Gene expression in human embryonic stem cell lines: unique molecular signature. Blood.

[B40] Skottman H, Mikkola M, Lundin K, Olsson C, Stromberg AM, Tuuri T, Otonkoski T, Hovatta O, Lahesmaa R (2005). Gene expression signatures of seven individual human embryonic stem cell lines. Stem Cells.

[B41] Adjaye J, Huntriss J, Herwig R, BenKahla A, Brink TC, Wierling C, Hultschig C, Groth D, Yaspo ML, Picton HM, Gosden RG, Lehrach H (2005). Primary differentiation in the human blastocyst: comparative molecular portraits of inner cell mass and trophectoderm cells. Stem Cells.

[B42] Babaie Y, Herwig R, Greber B, Brink TC, Wruck W, Groth D, Lehrach H, Burdon T, Adjaye J (2006). Analysis of OCT4 dependent transcriptional networks regulating self renewal and pluripotency in human embryonic stem cells. Stem Cells.

[B43] Bhattacharya B, Cai J, Luo Y, Miura T, Mejido J, Brimble SN, Zeng X, Schulz TC, Rao MS, Puri RK (2005). Comparison of the gene expression profile of undifferentiated human embryonic stem cell lines and differentiating embryoid bodies. BMC Dev Biol.

[B44] Yang D, Buchholz F, Huang Z, Goga A, Chen CY, Brodsky FM, Bishop JM (2002). Short RNA duplexes produced by hydrolysis with Escherichia coli RNase III mediate effective RNA interference in mammalian cells. Proc Natl Acad Sci U S A.

[B45] Zaehres H, Lensch MW, Daheron L, Stewart SA, Itskovitz-Eldor J, Daley GQ (2005). High-efficiency RNA interference in human embryonic stem cells. Stem Cells.

[B46] Hyslop L, Stojkovic M, Armstrong L, Walter T, Stojkovic P, Przyborski S, Herbert M, Murdoch A, Strachan T, Lako M (2005). Downregulation of NANOG induces differentiation of human embryonic stem cells to extraembryonic lineages. Stem Cells.

[B47] Damjanov I, Horvat B, Gibas Z (1993). Retinoic acid-induced differentiation of the developmentally pluripotent human germ cell tumor-derived cell line, NCCIT. Lab Invest.

[B48] Strumpf D, Mao CA, Yamanaka Y, Ralston A, Chawengsaksophak K, Beck F, Rossant J (2005). Cdx2 is required for correct cell fate specification and differentiation of trophectoderm in the mouse blastocyst. Development.

[B49] Kuhn K, Baker SC, Chudin E, Lieu MH, Oeser S, Bennett H, Rigault P, Barker D, McDaniel TK, Chee MS (2004). A novel, high-performance random array platform for quantitative gene expression profiling. Genome Res.

[B50] Jackson AL, Bartz SR, Schelter J, Kobayashi SV, Burchard J, Mao M, Li B, Cavet G, Linsley PS (2003). Expression profiling reveals off-target gene regulation by RNAi. Nat Biotechnol.

[B51] Kittler R, Buchholz F (2005). Functional genomic analysis of cell division by endoribonuclease-prepared siRNAs. Cell Cycle.

[B52] Hay DC, Sutherland L, Clark J, Burdon T (2004). Oct-4 knockdown induces similar patterns of endoderm and trophoblast differentiation markers in human and mouse embryonic stem cells. Stem Cells.

[B53] Teshima S, Shimosato Y, Hirohashi S, Tome Y, Hayashi I, Kanazawa H, Kakizoe T (1988). Four new human germ cell tumor cell lines. Lab Invest.

[B54] Russ AP, Wattler S, Colledge WH, Aparicio SA, Carlton MB, Pearce JJ, Barton SC, Surani MA, Ryan K, Nehls MC, Wilson V, Evans MJ (2000). Eomesodermin is required for mouse trophoblast development and mesoderm formation. Nature.

[B55] Scott IC, Anson-Cartwright L, Riley P, Reda D, Cross JC (2000). The HAND1 basic helix-loop-helix transcription factor regulates trophoblast differentiation via multiple mechanisms. Mol Cell Biol.

[B56] Firulli AB, McFadden DG, Lin Q, Srivastava D, Olson EN (1998). Heart and extra-embryonic mesodermal defects in mouse embryos lacking the bHLH transcription factor Hand1. Nat Genet.

[B57] Androutsellis-Theotokis A, Leker RR, Soldner F, Hoeppner DJ, Ravin R, Poser SW, Rueger MA, Bae SK, Kittappa R, McKay RD (2006). Notch signalling regulates stem cell numbers in vitro and in vivo. Nature.

[B58] Takahashi K, Yamanaka S (2006). Induction of Pluripotent Stem Cells from Mouse Embryonic and Adult Fibroblast Cultures by Defined Factors. Cell.

[B59] Loh YH, Wu Q, Chew JL, Vega VB, Zhang W, Chen X, Bourque G, George J, Leong B, Liu J, Wong KY, Sung KW, Lee CW, Zhao XD, Chiu KP, Lipovich L, Kuznetsov VA, Robson P, Stanton LW, Wei CL, Ruan Y, Lim B, Ng HH (2006). The Oct4 and Nanog transcription network regulates pluripotency in mouse embryonic stem cells. Nat Genet.

[B60] Dennis G, Sherman BT, Hosack DA, Yang J, Gao W, Lane HC, Lempicki RA (2003). DAVID: Database for Annotation, Visualization, and Integrated Discovery. Genome Biol.

[B61] Nishimoto M, Miyagi S, Yamagishi T, Sakaguchi T, Niwa H, Muramatsu M, Okuda A (2005). Oct-3/4 maintains the proliferative embryonic stem cell state via specific binding to a variant octamer sequence in the regulatory region of the UTF1 locus. Mol Cell Biol.

[B62] Vallier L, Reynolds D, Pedersen RA (2004). Nodal inhibits differentiation of human embryonic stem cells along the neuroectodermal default pathway. Dev Biol.

[B63] Ludwig TE, Levenstein ME, Jones JM, Berggren WT, Mitchen ER, Frane JL, Crandall LJ, Daigh CA, Conard KR, Piekarczyk MS, Llanas RA, Thomson JA (2006). Derivation of human embryonic stem cells in defined conditions. Nat Biotechnol.

[B64] Beattie GM, Lopez AD, Bucay N, Hinton A, Firpo MT, King CC, Hayek A (2005). Activin A maintains pluripotency of human embryonic stem cells in the absence of feeder layers. Stem Cells.

[B65] Xiao L, Yuan X, Sharkis SJ (2006). Activin a maintains self-renewal and regulates fibroblast growth factor, wnt, and bone morphogenic protein pathways in human embryonic stem cells. Stem Cells.

[B66] Amit M, Shariki C, Margulets V, Itskovitz-Eldor J (2004). Feeder layer- and serum-free culture of human embryonic stem cells. Biol Reprod.

[B67] Kim HJ, Bar-Sagi D (2004). Modulation of signalling by Sprouty: a developing story. Nat Rev Mol Cell Biol.

[B68] Greber B, Lehrach H, Adjaye J (2006). FGF2 Modulates TGF{beta} Signaling in MEFs and Human ES cells to Support hESC Self-renewal. Stem Cells.

[B69] Mohammadi M, McMahon G, Sun L, Tang C, Hirth P, Yeh BK, Hubbard SR, Schlessinger J (1997). Structures of the tyrosine kinase domain of fibroblast growth factor receptor in complex with inhibitors. Science.

[B70] Zaragosi LE, Ailhaud G, Dani C (2006). Autocrine FGF2 signaling is critical for self-renewal of Human Multipotent Adipose-Derived Stem Cells. Stem Cells.

[B71] Dvorak P, Dvorakova D, Koskova S, Vodinska M, Najvirtova M, Krekac D, Hampl A (2005). Expression and potential role of fibroblast growth factor 2 and its receptors in human embryonic stem cells. Stem Cells.

[B72] Kang HB, Kim JS, Kwon HJ, Nam KH, Youn HS, Sok DE, Lee Y (2005). Basic fibroblast growth factor activates ERK and induces c-fos in human embryonic stem cell line MizhES1. Stem Cells Dev.

[B73] Kittler R, Heninger AK, Franke K, Habermann B, Buchholz F (2005). Production of endoribonuclease-prepared short interfering RNAs for gene silencing in mammalian cells. Nat Methods.

[B74] Xu C, Inokuma MS, Denham J, Golds K, Kundu P, Gold JD, Carpenter MK (2001). Feeder-free growth of undifferentiated human embryonic stem cells. Nat Biotechnol.

[B75] Gene Expression Omnibus. http://www.ncbi.nlm.nih.gov/geo.

[B76] DAVID. http://david.abcc.ncifcrf.gov.

[B77] Chow S, Ruskey F, Liotta G (2004). Drawing Area-Proportional Venn and Euler Diagrams. Proceedings of Graph Drawing 2003.

