# Supplementary figures and images for: Silencing of core transcription factors in human EC cells highlights the importance of autocrine FGF signaling for self-renewal
Source: BMC Dev Biol. 2007 May 16;7:46. doi: 10.1186/1471-213X-7-46 (PMC1885259; doi:10.1186/1471-213X-7-46)

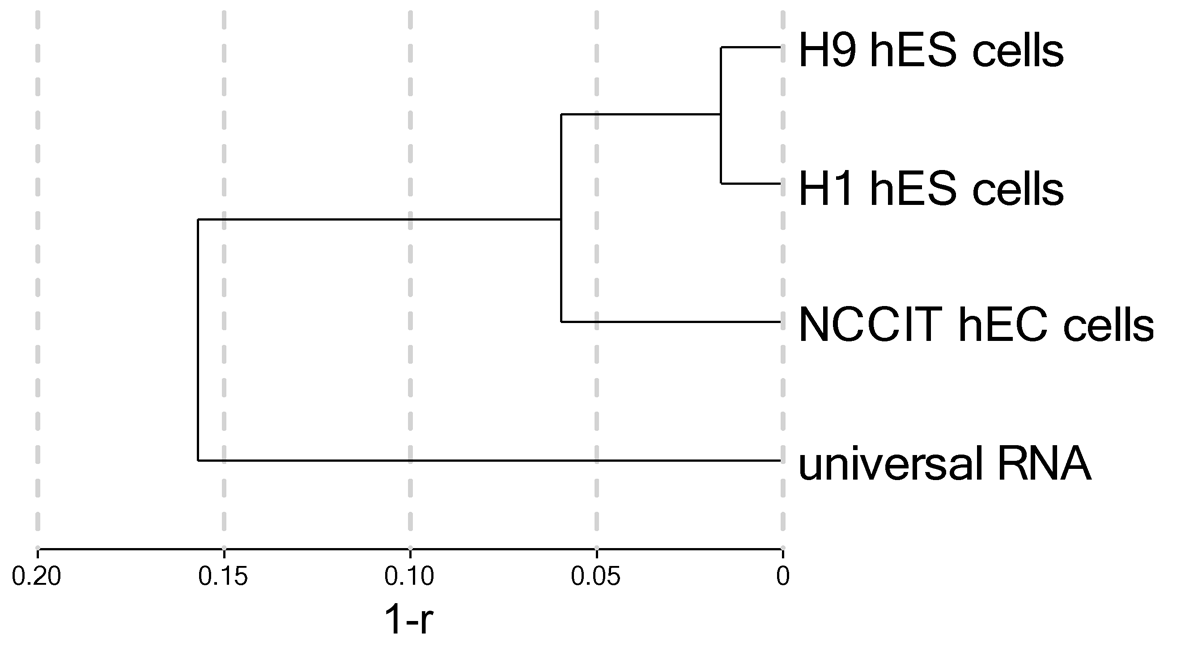

Supplement: Additional file 1 — Microarray-based transciptome comparison between hES cells and NCCIT hEC cells. Linear correlation coefficients (r) were 0.947 (NCCIT vs. H1), 0.934 (NCCIT vs. H9), and 0.984 (H1 vs. H9). Universal reference RNA (Stratagene) served as a baseline to identify genes preferentially expressed in hES and hEC cells. [file 1471-213X-7-46-S1.png]

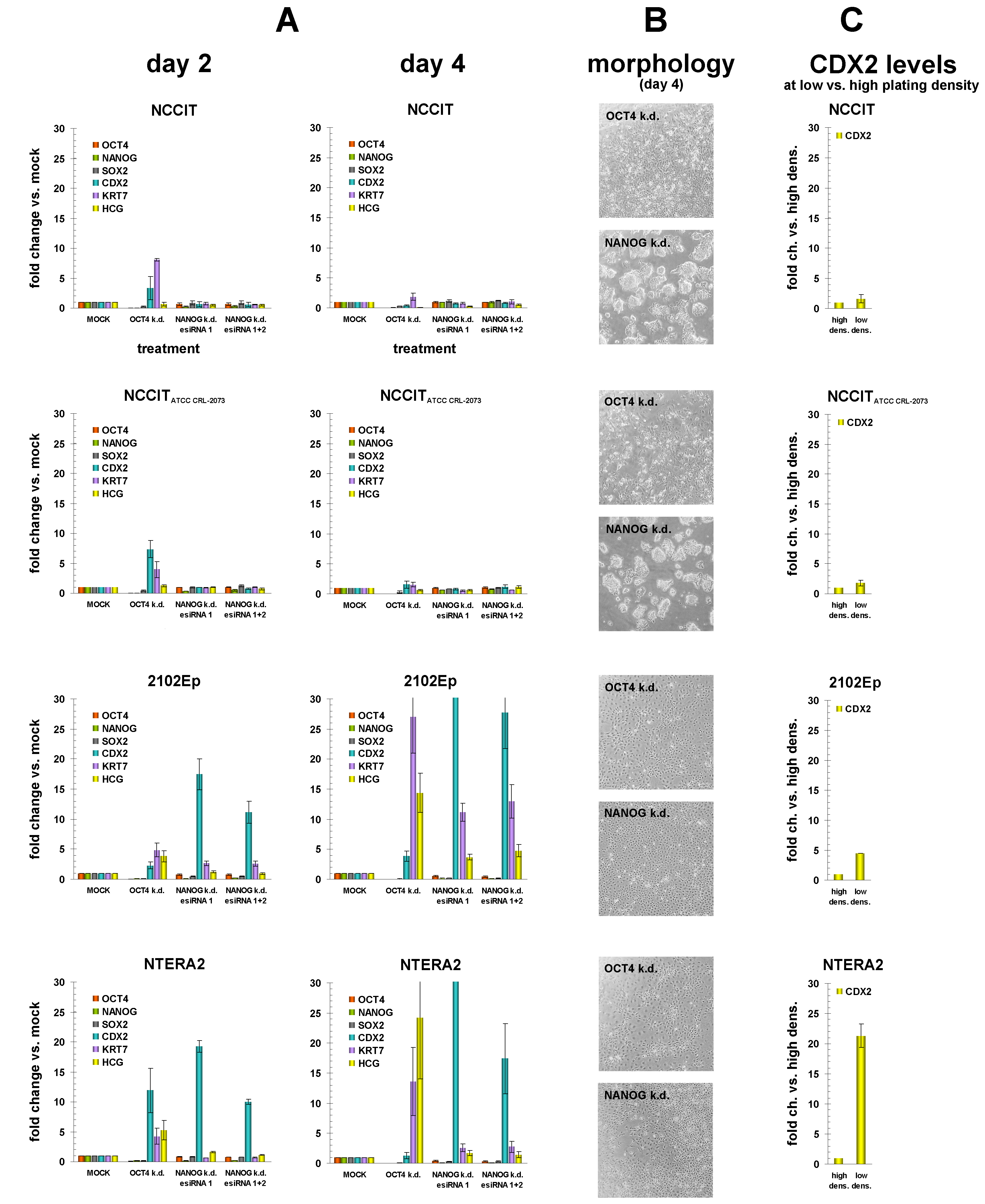

Supplement: Additional file 2 — Silencing of OCT4 and NANOG in 2102Ep, NTERA2, and two batches of NCCIT cells. (A) Real-time PCRs at two timepoints after esiRNA transfection using OCT4, NANOG, and SOX2 primers as well as markers of trophoblast differentiation. Bars indicate standard errors between two independent experiments (NCCIT) or between values based on distinct housekeeping controls (2102Ep and NTERA2). (B) Representative cellular morphology at day 4. See Fig. 3C for undifferentiated samples. (C) Real-time PCR-based comparison between cells kept undifferentiated by high density passaging vs. cells plated at low density as required for efficient gene silencing, using CDX2 as an early differentiation marker. [file 1471-213X-7-46-S2.png]

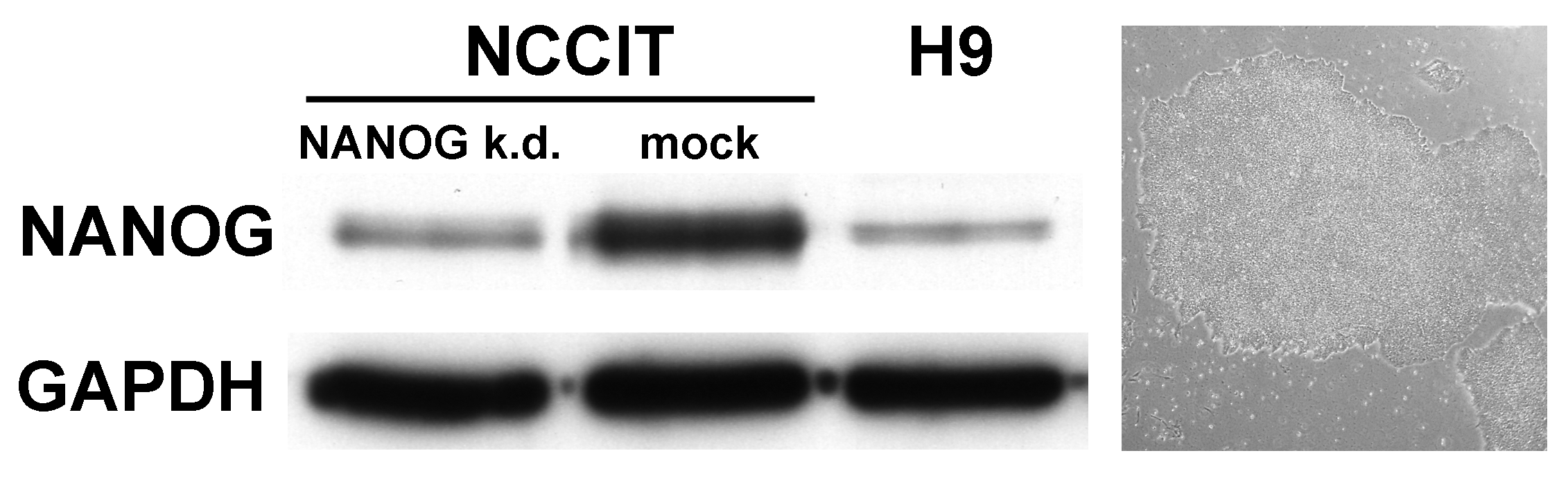

Supplement: Additional file 3 — NANOG protein levels in NCCIT (with and without transfection of NANOG esiRNA) and H9 hES cells. Left: Western blot probed with NANOG and GAPDH antibodies. Right: Undifferentiated morphology of hES cells prior to harvesting. [file 1471-213X-7-46-S3.png]

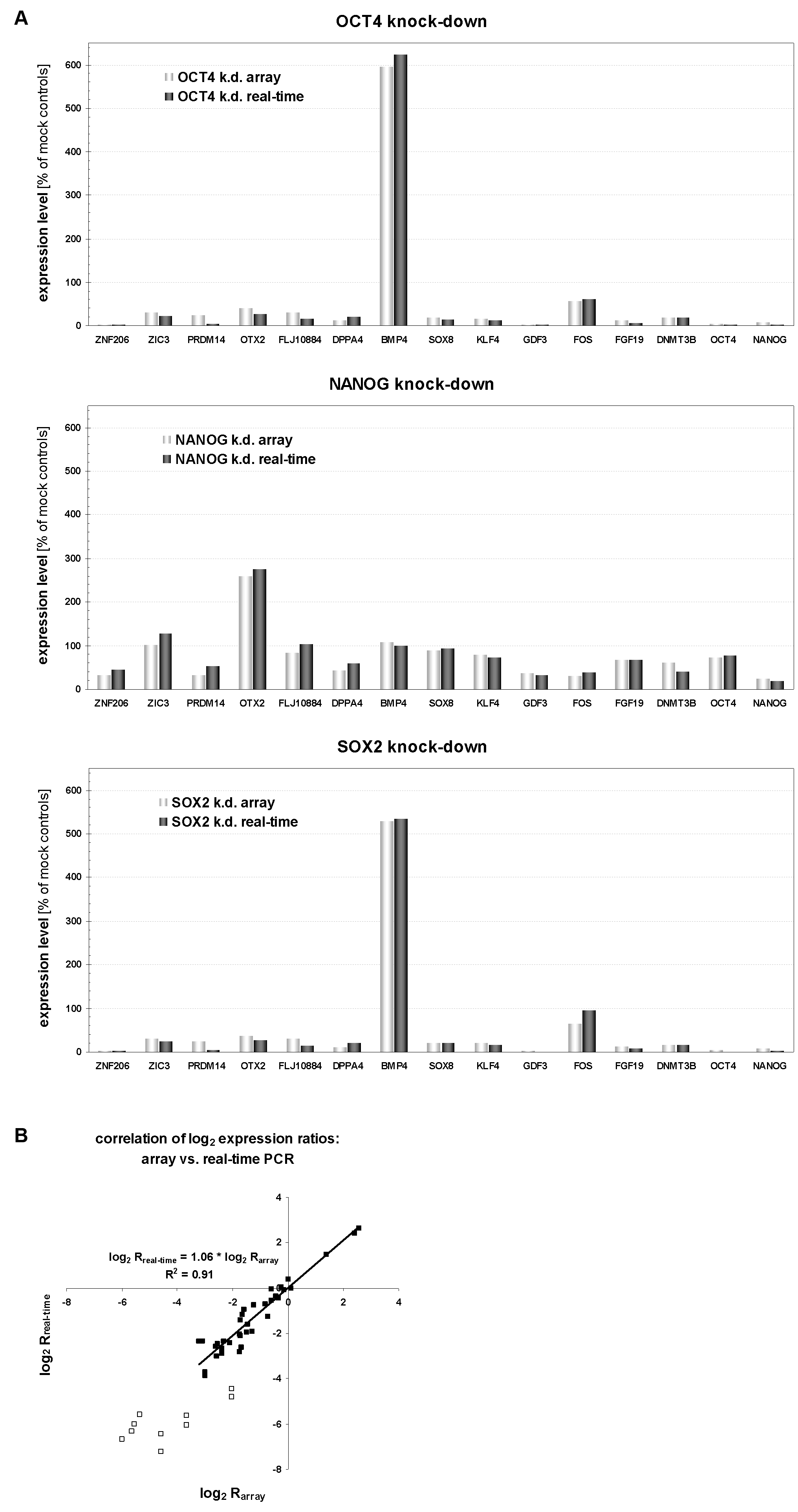

Supplement: Additional file 5 — Real-time PCR confirmations of differential gene expression in the OCT4, NANOG, and SOX2 RNAi samples. (A) Relative mRNA levels as measured with microarrays (light columns) and real-time PCR (dark columns). Real-time PCR measurements are based on two housekeeping control genes (GAPDH and ACTB). (B) Correlation between array and real-time PCR data of part A. The slope and correlation coefficient given refer to those transcripts that could reliably be detected by the arrays in both conditions (slope with all data points: 1.2; R2 = 0.88). [file 1471-213X-7-46-S5.png]
